# Prevalence of hs-CRP among Indians with hypertension

**DOI:** 10.6026/973206300181041

**Published:** 2022-10-31

**Authors:** Vasanthii R, Kaarthikeyan G, Sasirekha G, Mahalakshmi S

**Affiliations:** 1 Department of Biochemistry, Madurai Medical College,GRH, Madurai,India; 2 Saveetha Dental College & Hospital, Saveetha Institute of Medical and Technical Sciences (SIMATS), Chennai - 77, India

**Keywords:** Arterial hypertension, hs-CRP, atherosclerosis

## Abstract

The prevalence of hypertension in the early twentieth century varied in India, ranging from 2-15% in Urban India and 2-8% in Rural India. In the inter heart and inter stroke study, hypertension accounted for 17.9% and 34.6% of population attributable risk for coronary artery disease and stroke respectively. CRP appears in serum in response to a variety of inflammatory stimuli .Raised level of hs-CRP is seen with increasing with age, during an infection, inflammation, coronary artery diseases, obesity, sepsis, smoking and vasculitis. CRP is also a factor in the development of atherosclerotic plaque. Although CRP was believed to be a marker of vascular inflammation, recent research indicates that it plays an active role in atherogenesis. So in this study we measured serum hs- CRP in patients with essential hypertension and correlated with blood pressure. The study consists of 50 patients with essential hypertension with antihypertensive medications. in the age group of 40 to 60 years of both sexes and 25 normotensive subjects with no history of cardiovascular, neoplastic, hepatic, renal, infectious or auto immune disease. IHEC clearance and informed consent were obtained. hs-CRP was measured by ELISA kit. Our study showed significantly elevated serum hs-CRP level in hypertensive subjects in comparison with control subjects. To find out the relationship between physiological and biochemical parameters with CRP Pearson correlation coefficient has been applied. The level of significance has been fixed as 5% (p<0.05). SPSS15 software has been used for calculation. Our study showed significantly elevated serum hs-CRP level in hypertensive subjects in comparison with control subjects. But there is no correlation of hs-CRP level with both systolic and diastolic pressure. Several studies have shown inflammatory markers such as CRP as an independent determinant of endothelium dependent vascular function among patient with coronary heart disease (CHD) in patients with hypertension. There was no significant correlation was observed between levels of hs-CRP and systolic and diastolic blood pressure in this study but there was a significant elevation hs-CRP level was observed in hypertensive patients.

## Background:

A Hypertension is a commonly occurring, readily detectable disease. Arterial hypertension is a silent killer and major risk factor for atherosclerosis, coronary artery disease, stroke, kidney failure [1- 2]. The prevalence of hypertension in the early twentieth century varied in India, ranging from 2-15% in Urban India and 2-8% in Rural India. In the INTERHEART and INTERSTROKE study, hypertension accounted for 17.9% and 34.6% of population attributable risk for coronary artery disease and stroke respectively.CRP appears in serum in response to a variety of inflammatory stimuli .Raised level of hs-CRP is seen with increasing with age, during an infection, inflammation, coronary artery diseases, obesity, sepsis, smoking and vasculitis. Chronic vascular inflammation plays a role in initiation and the development of essential hypertension either as pathogenic or secondary event. Inflammatory mediators such as CRP, IL-1β, IL-6, TNF–α and reactive oxygen species have been proposed to contribute essential hypertension through several mechanism including enhancement of arterial stiffness, endothelial dysfunction [3]. hs-CRP is involved in vascular inflammation and plays a crucial role in the progression and development of atherosclerosis. Therefore, it is of interest to evaluate the relationship of serum high sensitive C-reactive protein (hs-CRP) and blood pressure in primary hypertensives.

## Material & Methods:

We enrolled 50 patients (male-17, female-33) with essential hypertension on medications without any complications in the age group of 40 to 60 years and 25 (male-8, female-17) normo tensive, healthy subjects. Institutional Human ethics committee clearance and informed written consent were obtained. Fasting blood samples were collected from the hypertensive patients and controls and analyzed for hematological and lipid, renal biochemical parameters by auto analyzer. Serum hs-CRP was assayed by ELISA kit.

### Statistical analysis:

Statistical analysis was done by Mann-Whitney U test using SPSS software and the level of significance was fixed at < 0.05. To find out the relationship between physiological and biochemical parameters with CRP Pearson correlation coefficient has been applied.

## Results:

There was no significant difference in BMI, plasma glucose, urea, creatinine and liver function tests in hypertensive subjects compared to controls. There was significant increase in total cholesterol (p< 0.05), Triglycerides (p < 0.001) and LDL-C (<0.05) in hypertensive subjects compared to controls. Serum hs-CRP level (9.06 ± 3.67 vs 3.74± 1.25) (p<0.001) was significantly increased in hypertensive subjects compared to controls.

## Discussion:

Serum hs-CRP was significantly increased in hypertensives in comparison with controls. CRP increases the blood pressure by several mechanisms. CRP inhibits formation of nitric oxide by endothelial cells which in turn promote vasoconstriction, leukocyte adhesion, platelet activation, oxidation and thrombosis [4]. High levels of CRP increases expression of endothelin-1 [5], and enhance expression of plasminogen activator inhibitor-1 by endothelial cells to promote vasoconstriction, platelet activation and thrombosis [6]. CRP has shown to upregulate angiotensin receptors thus enhancing angiotensin-II induced rise in blood pressure [7]. Angiotensin-II responsible for vascular inflammation by inducing oxidative stress, resulting in up-regulation of pro-inflammatory transcription factors such as NF-κB (nuclear factor κB). These, in turn, regulate the generation of inflammatory mediators that lead to endothelial dysfunction and vascular injury. Inflammatory markers (e.g. C-reactive protein, chemokines and adhesion molecules) are increased in patients with hypertension and predict the development of cardiovascular disease. In the study, even though hypertensive subjects were on antihypertensive drugs, there was a significant elevation of CRP levels found which suggesting the increased risk for cardio vascular complications? But there is no correlation of hs-CRP with systolic and diastolic blood pressure. Ki Chul Sung et al. found hs-CRP to be an independent risk factor for development of hypertension in Korean population [8]. In the year 2001, a cross-sectional study conducted by Bautista et al, for the first time measured CRP in hypertension and found CRP to be an independent risk factor for the development of hypertension [9]. In a study conducted by Bautista et al in 2003 did not find any association of hs-CRP with hypertension. They attributed this to the small sample size of their study [10]. Sesso et al. found a positive association between increasing levels of CRP and risk of developing hypertension. Though Sesso et al. suggested that higher hs-CRP were more likely to develop hypertension [11]. In the Strong Heart Study (2006), abnormal lipid profile (decrease in HDL cholesterol from baseline) was found to predict development of hypertension in American Indian population in 8 year follow up [5]. In the CARDIA study, development of incident hypertension was associated with initial systolic BP, levels of triglycerides and HDL-cholesterol over 10 years in 5115 black and white young adults [6].Marco et al in 2009 (the strong heart study data) study found that those pre-hypertensives who developed hypertension had higher levels of inflammatory markers, higher triglycerides and lower HDL cholesterol [12].

## Conclusions:

Though hs-CRP increased in hypertension, it is not associated with disease process. The influencing of aging and dyslipidemia involved in progressing inflammatory and degenerative process, on serum hs-CRP level could not ruled out.
